# Label-free optical biomarkers detect early calcific aortic valve disease in a wild-type mouse model

**DOI:** 10.1186/s12872-020-01776-8

**Published:** 2020-12-11

**Authors:** Ishita Tandon, Shelby Johns, Alan Woessner, Jessica Perez, Delaney Cross, Asya Ozkizilcik, Timothy J. Muldoon, Srikanth Vallurupalli, Muralidhar Padala, Kyle P. Quinn, Kartik Balachandran

**Affiliations:** 1 Department of Biomedical Engineering, University of Arkansas, 122 John A. White Jr. Engineering Hall, Fayetteville, AR 72701 USA; 2 Division of Cardiology, University of Arkansas for Medical Sciences, Little Rock, AR 72205 USA; 3 Division of Cardiothoracic Surgery, Joseph P. Whitehead Department of Surgery, Emory University, Atlanta, GA 30322 USA

**Keywords:** Calcific aortic valve disease, Valve calcification, Two-photon excited fluorescence microscopy, Wild-type mouse model

## Abstract

**Background:**

Calcific aortic valve disease (CAVD) pathophysiology is a complex, multistage process, usually diagnosed at advanced stages after significant anatomical and hemodynamic changes in the valve. Early detection of disease progression is thus pivotal in the development of prevention and mitigation strategies. In this study, we developed a diet-based, non-genetically modified mouse model for early CAVD progression, and explored the utility of two-photon excited fluorescence (TPEF) microscopy for early detection of CAVD progression. TPEF imaging provides label-free, non-invasive, quantitative metrics with the potential to correlate with multiple stages of CAVD pathophysiology including calcium deposition, collagen remodeling and osteogenic differentiation.

**Methods:**

Twenty-week old C57BL/6J mice were fed either a control or pro-calcific diet for 16 weeks and monitored via echocardiography, histology, immunohistochemistry, and quantitative polarized light imaging. Additionally, TPEF imaging was used to quantify tissue autofluorescence (A) at 755 nm, 810 nm and 860 nm excitation, to calculate TPEF 755–860 ratio (A_860/525_/(A_755/460_ + A_860/525_)) and TPEF Collagen-Calcium ratio (A_810/525_/(A_810/460_ + A_810/525_)) in the murine valves. In a separate experiment, animals were fed the above diets till 28 weeks to assess for later-stage calcification.

**Results:**

Pro-calcific mice showed evidence of lipid deposition at 4 weeks and calcification at 16 weeks at the valve commissures. The valves of pro-calcific mice also showed positive expression for markers of osteogenic differentiation, myofibroblast activation, proliferation, inflammatory cytokines and collagen remodeling. Pro-calcific mice exhibited lower TPEF autofluorescence ratios, at locations coincident with calcification, that correlated with increased collagen disorganization and positive expression of osteogenic markers. Additionally, locations with lower TPEF autofluorescence ratios at 4 and 16 weeks exhibited increased calcification at later 28-week timepoints.

**Conclusions:**

This study suggests the potential of TPEF autofluorescence metrics to serve as a label-free tool for early detection and monitoring of CAVD pathophysiology.

## Background

Calcific aortic valve disease (CAVD) is the most common form of valvulopathy in the western world, with a prevalence of 13.3% in people above 75 years of age [1, 2]. CAVD is associated with a 50% elevated risk of morbidity and mortality [3]. Once severe aortic stenosis develops, valve replacement, either via surgical or transcatheter approaches, is the current treatment standard, as early detection, prevention, and mitigation strategies have yet to be clinically adopted [1, 2, 4]. There is thus a need to develop better diagnostic and therapeutic approaches for CAVD.

The early pathogenic processes in CAVD are defined by the phenotypic transformation of valve endothelial (VEC) and interstitial (VIC) cells via cellular activation, osteogenic differentiation [5–7], as well as maladaptive extracellular matrix (ECM) remodeling [8, 9]. CAVD pathophysiology is marked by infiltration of cytokines, lipid deposition, and calcific nodule formation [4, 10, 11]. These early pathogenic processes are simulated using two- and three-dimensional in vitro models and in vivo models that mimic the CAVD process and valve milieu to varying degrees of accuracy [7, 12–15]. Wild-type diet-based mouse CAVD models have previously demonstrated deposition of monocyte-macrophages, lipids, and lipoproteins [16, 17], but to our knowledge no such diet-based models exhibit early calcification as demonstrated by positive Alizarin Red S (ARS) staining, as well as positive osteopontin and Runt-related transcription factor 2 (RUNX2) expression [14,17]. Assmann and colleagues have shown that a diet regimen supplemented with vitamin D, cholesterol and dicalcium phosphate caused significant calcification of the aortic root in Wistar rats [18]. Vitamin D in combination with cholesterol has been separately shown to increase vascular calcification in rats [19]. Vitamin D is responsible for maintaining serum calcium levels in humans and both deficiency and excess of Vitamin D has been shown to promote cardiovascular calcification [20–22]. Higher calcium phosphate and elevated serum calcium are also established risk factors for calcification progression [21, 22]. We exploited a similar dietary regimen to develop a wild-type mouse model for early CAVD.

Multiple biochemical and imaging markers are associated with independent events such as endothelial dysfunction, inflammatory cytokine infiltration, matrix remodeling, and mineral deposition to assess either the presence or severity of calcification [2, 4, 10, 11, 23]. However, there is a dearth of label-free imaging biomarkers to monitor the progression of calcification in the aortic valve, both in clinics and research laboratories [23]. Clinically, a stenotic aortic valve usually is detected by auscultation of a systolic murmur and confirmed by echocardiography, and relies on late hallmarks of CAVD like hemodynamic malfunction and impaired geometry of the valve [24]. Imaging techniques such as cardiac magnetic resonance imaging and cardiac computed tomography have also been shown to be sensitive to the late hallmarks of the disease; however, there is a specific lack of tools to detect early CAVD progression [24–26]. Two-photon excited fluorescence (TPEF) microscopy has recently shown potential in providing label-free quantitative metrics that might associate with osteogenic differentiation [27]. TPEF allows quantification of the endogenous fluorescence ratios of the cellular co-factors flavin adenine (FAD) and nicotinamide adenine (NADH) dinucleotides in their oxidized and reduced forms, respectively and is known as the optical redox ratio (i.e. FAD/(FAD + NADH)) [27]. It was previously established in vitro that osteogenic differentiation of mesenchymal stem cells is associated with a reduction in TPEF-derived optical redox ratios [27]. We have also demonstrated that this TPEF-based optical redox ratios in VICs was reduced as cellular stretching increased from normal to pathologic magnitudes [13]. Additionally, optical redox ratio altered and correlated with temporal changes in VIC phenotype during osteogenic de-differentiation, in vitro [28]. In addition to NADH and FAD, molecules such as collagen, calcium and lipids within tissues will contribute to the autofluorescence emission in the visible range [27, 29–32]. Recently, TPEF has been used to identify endogenous fluorescence from calcium deposition that correlated with mineralization in ApoE^−/−^mice and calcified human valves [29].

In this study, based on the above prior work, we hypothesized that TPEF autofluorescence markers would coincide with traditional phenotypic markers that indicate early progression of CAVD. We tested this hypothesis using ex vivo imaging of valves from a diet-based wild-type C57BL/6J mouse model that demonstrated early CAVD progression with lipid and calcium deposition, and matrix remodeling. In our model, the aortic valve commissures showed lower TPEF autofluorescence ratios, which was concurrent with increased cell activation, inflammatory cytokine expression, cell proliferation and markers for osteogenic differentiation, and which was predictive of increased calcification at later time points.

## Methods

### Experimental animals for early-CAVD model

All animal experiments were performed in compliance with the University of Arkansas Institutional Animal Care and Use Committee (IACUC) approval and conform to all appropriate guidelines. Male wild-type C57BL/6J mice were obtained from The Jackson Laboratory (Bar Harbor, ME) and housed at most four per cage under optimum temperature, humidity and light cycle. Adult mice aged 20 weeks [33] were randomized and fed an open standard diet, with either a control or pro-calcific diet supplementation (Research Diets, New Brunswick, NJ). The differences in the dietary supplements [18] are outlined in Table 1. The amount of food intake was monitored per cage and the mice were weighed every three to four days till 16 weeks. In a separate experiment, animals were fed the above diets till 28 weeks to assess for later-stage calcification.

**Table 1:** Differences in supplementation for control and pro-calcific diets

	Control diet	Pro-calcific diet
Cholesterol	0.0% (w/w)	2.07% (w/w)
Total Vitamin D3	934 (IU/kg)	75,042 (IU/kg)
Calcium	0.57% (w/w)	0.99% (w/w)
Phosphorus	0.435% (w/w)	0.75% (w/w)

### Echocardiography for animal model

Echocardiography was performed for all the mice in the study using standard protocols [5]. At the 4, 8, 12, 16 and 28-week time points, mice were anesthetized using inhalative 4% isoflurane with 2 L/Min oxygen. Mice were maintained at 1% isoflurane with 2 L/Min oxygen through a nose cone on a heated pad for the entire echocardiography procedure. After hair removal from the ventral side, an Agilent SONOS 5500 (Agilent Technologies, Santa Clara, CA) ultrasound machine was used to obtain and analyze 2D and M-mode images to measure the diastolic (LVIDd) and systolic (LVIDs) left ventricle internal dimensions. The heart rate (HR) was monitored and recorded simultaneously via Covidien Kendall 230 conductive adhesive hydrogel electrodes (Dublin, Ireland). Ejection Fraction (EF), End Diastolic Volume (EDV), End Systolic Volume (ESV) and Cardiac Output (CO) were assessed using Simpson’s Method, via the below Eqs. (5). At least seven animals per condition per time point were assessed.

$$EDV=\frac{7.0+LVID{d}^{3}}{2.4+LVIDd}$$

$$ESV=\frac{7.0+LVID{s}^{3}}{2.4+LVIDs}$$

$$EF(\%)=\frac{\left(LVID{d}^{3}-LVID{s}^{3}\right)\times 100}{LVID{d}^{3}}$$

$$CO=(EDV-ESV)\times HR$$

### Pathophysiological assessment and tissue processing

At the aforementioned time points, the animals were euthanized by cervical dislocation, and their hearts were extracted, washed thoroughly in cold, sterile PBS and frozen in optimum cutting temperature compound (Sakura Finetek USA Inc, Torrance, CA). Serial 5–8 µm transverse sections of the aortic valve were obtained using a Leica CM1860 cryotome (Buffalo grove, IL). Blood was collected in heparin coated tubes and plasma was stored at -80 freezer for further processing.

### Histology and Immunohistochemistry

Hematoxylin and eosin (H&E; IHC World, Woodstock, MD), Oil red O (Electron Microscopy Sciences, Hatfield, PA), Alizarin Red S (ARS; Sigma-Aldrich, St. Louis, MO) and Picrosirius Red (PSR; Electron Microscopy Sciences, Hatfield, PA) staining were performed using standard protocols [5, 18]. Oil red O was utilized to assess lipid deposition, ARS was used for detecting calcium deposition and PSR stain was used to analyze tissue collagen fibers. Immunohistochemistry was performed using primary antibodies (all from Abcam, Cambridge, MA) against αSMA (1:100), vimentin (1:300), RUNX2 (1:200), osteopontin (1:200), BMP4 (1:100), Ki67 (1:500), TGFβ1 (1:50). Appropriate Alexa fluor-488 and − 594 (1:200; Life Technologies, Carlsbad, CA) conjugated secondary antibodies were used for immunolabeling. Negative controls were labeled only with secondary antibodies (Additional file 1: Fig. 1) to assess nonspecific binding. A Nikon (Tokyo, Japan) epifluorescence microscope in conjunction with NIS Elements software was used to obtain both bright-field and fluorescent images. A Nikon C-SP simple polarizer eclipse (model no. MBB75370) was attached to the microscope and polarized light microscopy was utilized to image the PSR stained sections.

To quantify the ARS positive calcification, images were loaded onto Fiji-ImageJ and converted to 8-bit format. A constant threshold was applied and the plugin ‘Analyze Particles’ was used to output the percentage area positive for the ARS staining (Additional file 1: Fig. 2) in leaflets, commissures, and root separately by outlining as described for TPEF metrics in Additional file 1: Fig. 3D. Analysis of PSR stained sections was performed using a custom MATLAB code (MathWorks, Natick, MA) for the aortic root, commissures and leaflet regions separately [5]. Collagen fiber thickness was assessed based on color and binned into four categories—thick (red), thick intermediate (orange), thin intermediate (yellow) and thin (green) [5]. At least three to four biological replicates per group were analyzed. For immunohistochemistry, three individual researchers, blinded to the study, qualitatively assessed three biological replicates per group and provided a score of 0 for no or low expression, 1 for moderate expression, or 2 for high expression. Scores of the three individuals were averaged and assigned a “-” if scored as 0, “ + ” if the mean score was from 0—0.66, “ + + ” if scored from 0.66 to 1.33 or “ + + + ” if scored from 1.33 to 2. The averaged mean score from the three individuals was used to perform correlation analysis with TPEF metrics.

### Quantitative polarized light imaging

Quantitative polarized light imaging (QPLI) was used to image PSR stained sections, to measure collagen fiber orientation, and thickness through phase retardation. Briefly, a custom-built trans-illumination based QPLI microscope (Olympus BX51, Olympus Corp., Tokyo, Japan) setup with a rotating polarizer and circular analyzer was utilized [34–37]. Image collection was completed using a 20 × objective (UPlanFL N 20x, 0.5 NA, Olympus Corp.) in order to accommodate the size of the samples, and full field images were produced via image stitching. The images were analyzed using a custom MATLAB code for the aortic root, commissures and leaflet regions separately (Additional file 1: Fig. 3A). Average light retardation, which is proportional to the thickness of collagen at each pixel location, was assessed as the primary QPLI metric. A rotating polarizer and circular analyzer enabled the assessment of variance in collagen fiber direction at each pixel [36, 37], and directional variance of fiber orientations was computed to evaluate the relative strength of collagen fiber alignment in the mean direction [37, 38]. At least three to four biological replicates per group were analyzed.

### Two photon excited fluorescence (TPEF) imaging

TPEF imaging was carried out using a custom-built resonant-scanning setup (Thorlabs, Newton, NJ) with a Mai-Tai ultrafast Ti:Sapphire tunable laser source (Spectra-Physics, Santa Clara, CA), [13] with a (20x, 0.75 NA) water immersion objective (Nikon, Japan). Tissue sections utilized for TPEF imaging were frozen, unprocessed and unfixed. It has been previously shown that TPEF autofluorescence ratio of ex vivo frozen tissue sections correlates highly with in vivo measurements [39]. The average intensity of the region of interest at a given excitation and emission wavelength is represented as A_excitation/emission_. TPEF autofluorescence was collected at 755 nm excitation with 460 nm/40 nm emission for A_755/460_, 860 nm excitation with 525 nm/45 nm emission for A_860/525_ (Additional file 1: Fig. 3B), 810 nm excitation with 460 nm/40 nm emission for A_810/460_ and 525 nm/45 nm emission for A_810/525_ (Additional file 1: Fig. 3C) [27, 29]. Laser power of 40mW and photomultiplier tube (PMT) gain was kept consistent throughout imaging. Two TPEF autofluorescence metrics were calculated using a custom MATLAB code. The aortic root, commissures and leaflet regions were manually outlined and analyzed separately (Additional file 1: Fig. 3D). The following equations were utilized to obtain the autofluorescence intensity ratios at each pixel.

$${\text{TPEF 755 - 860 Ratio}} = \frac{{A_{860/525} }}{{A_{755/460} + A_{860/525} }}$$

$${\text{TPEF Collagen - Calcium (Col - Cal) Ratio}} = \frac{{A_{810/525} }}{{A_{810/460} + A_{810/525} }}$$

It should be noted that both these TPEF autofluorescence ratios are influenced by the collagen, lipids, mineralized calcific deposits, NADH and FAD [29, 30]. TPEF 755–860 ratio has often been used as an optical redox ratio when NADH and FAD are the only significant fluorophores [27, 32, 40]. Four biological replicates were utilized for the TPEF imaging per experimental treatment group for the mice.

### Statistical analysis

All data was represented as mean ± standard error of the mean (SEM). Two-way analysis of variance (ANOVA) was utilized for comparison between the treatment groups at different time points for the murine samples with Tukey’s post-hoc tests if data passed normality tests. A p-value of less than 0.05 was considered statistically significant. The exact p-value is reported wherever relevant, if it was less than 0.1. The Kruskal–Wallis ANOVA on ranks non-parametric test was utilized if data did not pass the normality tests. Pearson’s correlation coefficient was used for the correlation analysis of normally distributed data and Spearman’s Rank correlation was used for the correlation analysis of non-parametric data including immunohistochemistry scoring. All scoring of immunohistochemistry images was performed in a blinded manner. JMP (SAS Institute, Cary, NC) and SigmaPlot (Systat Software Inc., San Jose, CA) were utilized for the data analysis and graphing.

## Results

### Mice on pro-calcific diet had lipid and calcium deposition at the aortic valve commissures

**Fig. 1:**
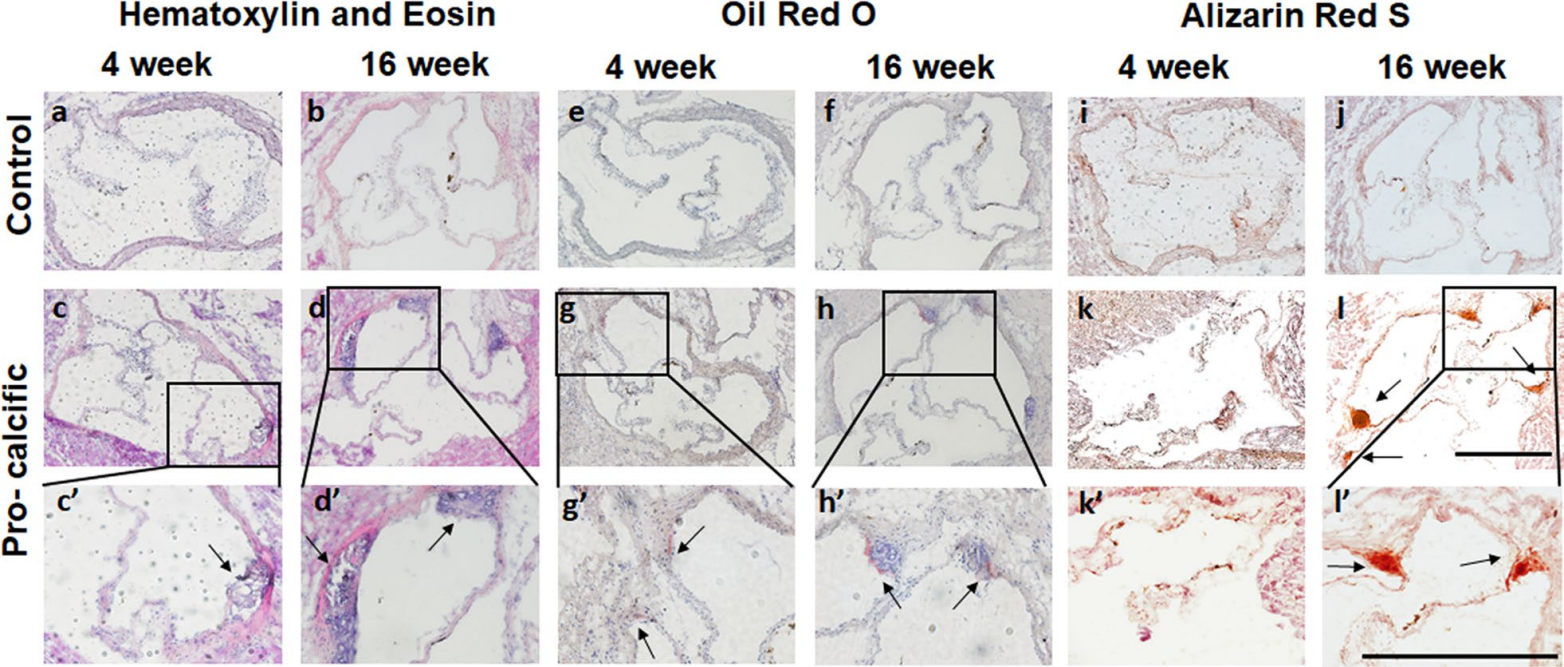
Lipid and calcium Deposition assessed by Histology. **a**–**d** Representative Hematoxylin and Eosin stained transverse sections at 10 ×, and **c**, **d** 20 × depicting plaque-like structures near the commissural walls in pro-calcific mice as marked by arrows. **e**–**h** Representative Oil Red O stained transverse sections at 10 ×, and **g**, **h** 20 × depicting lipid deposition at the commissural walls in pro-calcific mice as marked by arrows. **i**–**l** Representative Alizarin Red S (ARS) stained transverse sections at 10 ×, and **k**, **l** 20 × depicting calcium deposition at the commissural walls in pro-calcific mice at 16 weeks, as marked by arrows. Scale Bars—500 µm. N = 3 (mouse)

Histology was employed to assess the morphology of the aortic valve and typical hallmarks of CAVD progression such as lipid deposition and calcification [11, 17, 18, 41]. Hematoxylin and Eosin staining revealed plaque-like structures in the aortic root proximal to the commissures at 4 weeks in the mice fed a pro-calcific diet (Fig. 1a–d). Oil Red O staining was performed to confirm if the pro-calcific mice did indeed have deposition of lipid vacuoles. The control mice did not stain positive for Oil Red O (Fig. 1e, f), however, the pro-calcific mice showed positive Oil Red O staining at 4 weeks at the valve commissures (Fig. 1g, h). Qualitatively, no increase in Oil Red O-positive lipid deposition was observed in the pro-calcific groups at 16 weeks compared to the 4-week time point. Alizarin Red S (ARS) staining was performed to assess calcium deposition. Positive ARS staining was not observed in the control mice for all time points (Fig. 1i, j). Pro-calcific mice showed positive ARS staining in the commissures at the 16-week time point (Fig. 1k, l).

### Mice on pro-calcific diet had increased expression of markers for cell activation and osteogenic dedifferentiation

**Fig. 2:**
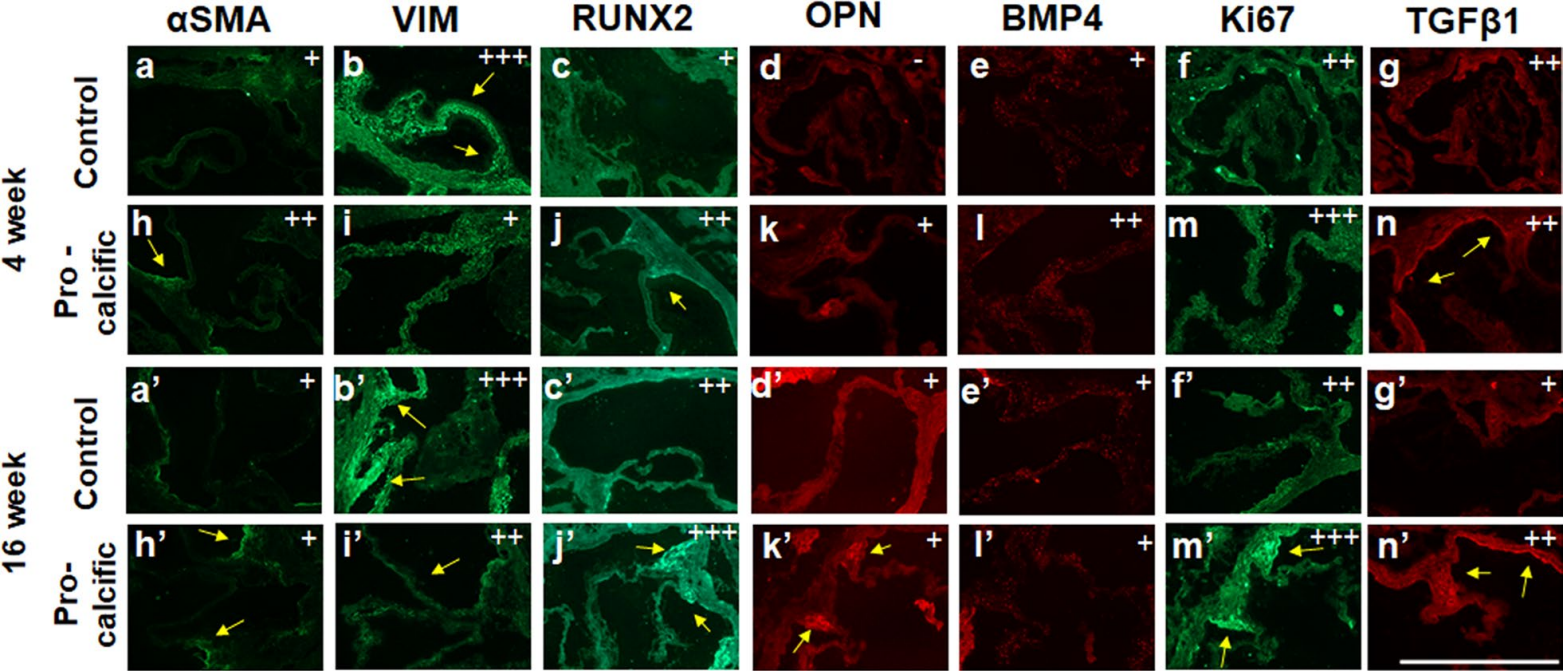
Expression of phenotypic and functional markers assessed by Immunohistochemistry. Representative transverse sections of aortic valves from control and pro-calcific mice at 4 weeks showing expression of **a**, **h** alpha smooth muscle actin (αSMA), **b**, **i** vimentin, **c**, **j** RUNX2, **d**, **k** osteopontin, **e**, **l** bone morphogenic protein 4 (BMP4), **f**, **m** Ki67 and **g**, **n** transforming growth factor β1 (TGFβ1). Representative transverse sections of aortic valves from control and pro-calcific mice at 16 weeks showing expression of **a**′, **h**′ alpha smooth muscle actin (αSMA), **b**′, **i**′ vimentin, **c**′, **j**′ RUNX2, **d**′, **k**′ osteopontin, **e**′, **l**′ bone morphogenic protein 4 (BMP4), **f**′, **m**′ Ki67 and **g**′, **n**′ transforming growth factor β1 (TGFβ1). Positive expression is marked by arrows and scored as -, + , + + or + + + . The stars denote autofluorescence from the calcified lesions. Scale Bars—500 µm. N = 3 (mice)

Immunohistochemistry was performed to further investigate the early and late markers of aortic valve disease progression [18, 42]. Control mice showed lower α-SMA expression and higher vimentin expression suggesting a quiescent phenotype of the valve cells under this treatment (Fig. 2a, b, a′, b′) [43]. Higher α-SMA and lower vimentin (Fig. 2h, i, h′, i′) expression was observed in the pro-calcific mice, suggesting an activated cell phenotype [43]. There was also higher osteopontin expression (Fig. 2d, k, d′, k′) at the commissures of pro-calcific mice as compared to control mice, suggesting an osteogenic phenotype in these samples [2, 18]. The expression of RUNX2 in pro-calcific mice was also qualitatively more pronounced at the 16-week time point (Fig. 2c′, j′) as compared to the 4-week time point (Fig. 2c, j) suggesting disease progression over the course of this timespan [2]. Osteopontin and BMP4 expression (Fig. 2e, l, e′, l′) showed an increase in pro-calcific mice only at 4 weeks suggesting early changes. Control mice had lower Ki67 expression as compared to pro-calcific mice at the commissures, suggesting that the valvular cells in the pro-calcific groups were more proliferative [5, 39] (Fig. 2f, m, f′ m′). TGFβ1, a marker for inflammatory cytokines [42], expressed positively at the commissures in pro-calcific mice (Fig. 2g, n, g′, n′) at 16 weeks.

### Mice on pro-calcific diet showed increased collagen remodeling compared to control diet

**Fig. 3:**
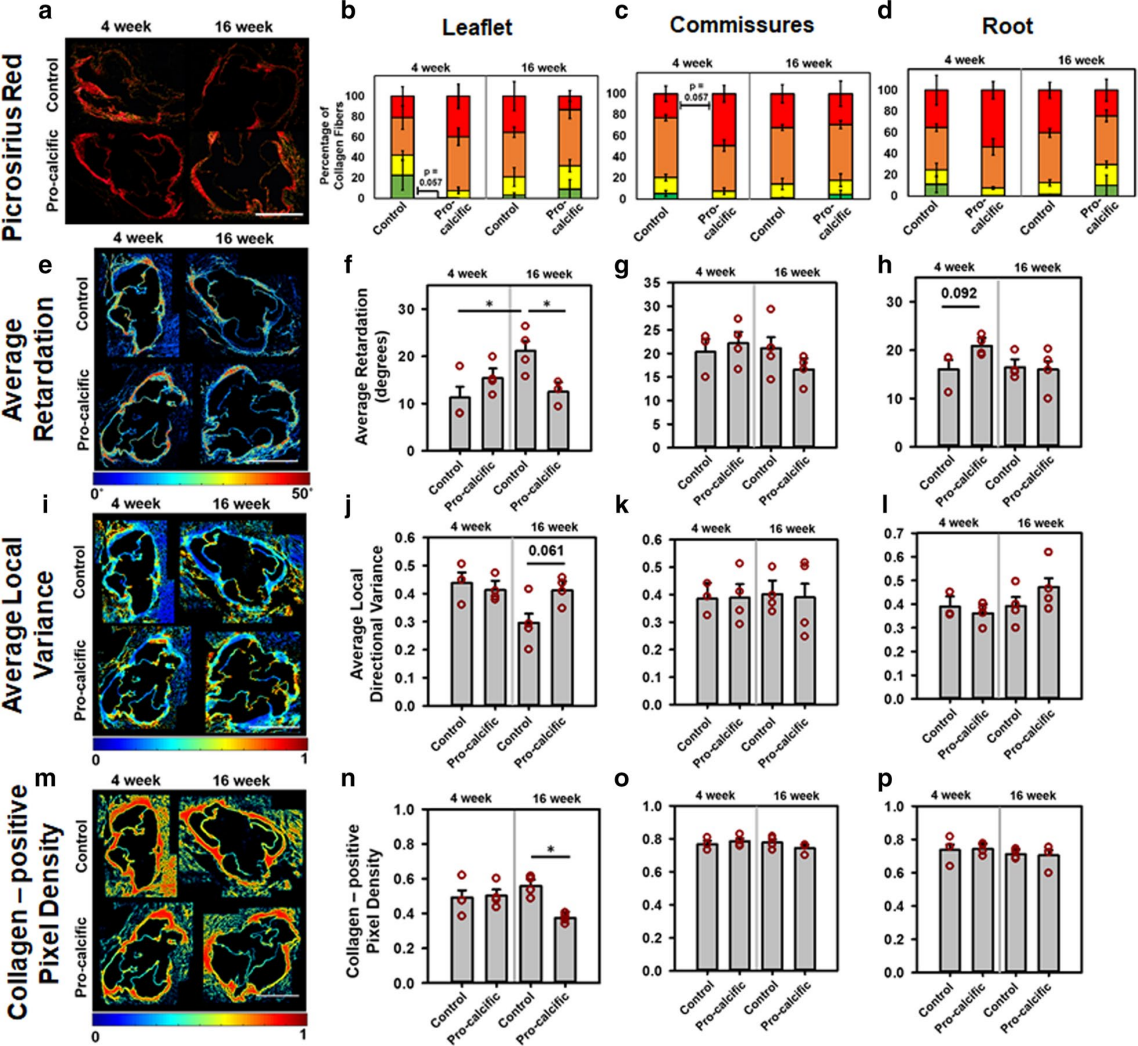
Assessment of collagen remodeling via PSR and QPLI. **a** Representative picrosirius red (PSR) stained aortic valves for control and pro-calcific mice at 4 and 16 weeks imaged by linear polarized light microscopy. Scale Bar—500 µm. Percentage of thin, intermediate and thick collagen fibers for the **b** leaflets, **c** commissures and **d** roots. **e** Representative images for quantitative polarized light imaging (QPLI) analyzed aortic valves for control and pro-calcific mice at 4 and 16 weeks depicting average retardation ranging from 0˚ to 50˚. Scale Bar—500 µm. Computed average retardation for **f** leaflets, **g** commissures and **h** roots. **i** Representative images for control and pro-calcific mice at 4 and 16 weeks depicting average local variance in the collagen fiber orientation. Scale Bar—500 µm. Computed average local variance for **j** leaflets, **k** commissures and **l** roots. **m** Representative images for control and pro-calcific mice at 4 and 16 weeks depicting average collagen positive pixel density. Computed average collagen positive pixel density for **n** leaflets, **o** commissures and **p** roots. Scale Bar—500 µm. N = 4. **p < 0.01, *p < 0.05. Mice PSR, average retardation, average local directional variance and collagen positive pixel density: Two-way ANOVA with Tukey HSD post hoc test

To further characterize structural markers for disease progression, we assessed collagen remodeling via PSR staining and quantitative polarized light imaging (QPLI) techniques. PSR staining was performed to enhance the birefringence of collagen [44] present in the mouse valves (Fig. 3a). The PSR stained sections were first imaged through a linear polarizer to analyze the thickness of collagen fibers, a metric that is usually associated with collagen fiber diameter, where the increase in thickness corresponds to a shift from green (thinner) to red (thicker) fibers. It is important to note that this shift from green to red is also dependent on the packing density and alignment of collagen fibers [45]. A lower percentage of thinner (green) fibers was observed in the leaflets (p = 0.057) (Fig. 3b) in the pro-calcific valves at 4 weeks as compared to the control valves. A higher percentage of thicker (red) fibers was observed in the commissures (p = 0.057) (Fig. 3c) in the pro-calcific valves at 4 weeks as compared to the control valves. However, at 16 weeks these differences disappeared. No differences were observed in fiber thickness between the roots of control versus pro-calcific mice (p > 0.1) (Fig. 3d).

Via QPLI, average retardation (Fig. 3e) showed a significant reduction in the leaflets of pro-calcific mice as compared to the control mice at 16 weeks (p = 0.0455) (Fig. 3f). Compared to the control mice at 4 weeks, the average retardation was significantly higher in control mice at 16 weeks (p = 0.0269). The commissures showed no statistically significant differences in the average retardation (p > 0.1) (Fig. 3g). The aortic root tended to have an increased average retardation in the pro-calcific group as compared to the control mice at 4 weeks (p = 0.092) (Fig. 3h).

Average local directional variance in the collagen fiber orientation was used as a metric to assess the alignment of collagen fibers (Fig. 3i) [38]. Average local directional variance increased in the leaflets of the pro-calcific mice as compared to the control mice at 16 weeks (p = 0.061) (Fig. 3j). There were no significant differences (p > 0.1) observed in the commissures (Fig. 3k) and roots (Fig. 3l).

Average collagen positive pixel density (Fig. 3m) was assessed as an additional parameter for collagen remodeling [38]. The leaflets showed significantly reduced collagen positive pixel density in the pro-calcific group at 16 weeks, as compared to the control mice at 16 weeks (p = 0.0147) and pro-calcific mice at 4 weeks (p = 0.0747) (Fig. 3n). There were no significant differences (p > 0.1) observed in the commissures (Fig. 3o) and roots (Fig. 3p).

### Changes in individual A_755/460,_ A_860/525,_ A_810/460_ and A_810/525_ autofluorescence intensities

**Fig. 4:**
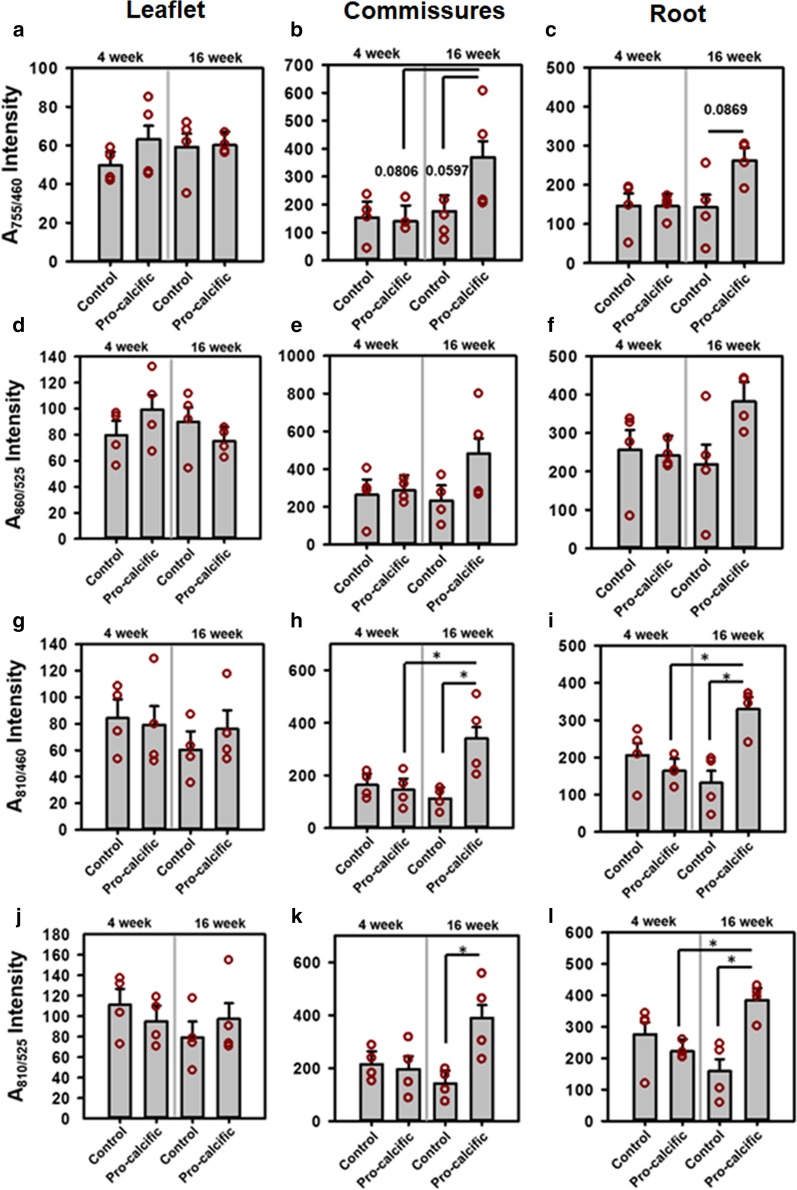
Average Intensities utilized to calculate TPEF Autofluorescence Ratios. Average A_755/460_ intensities of control and pro-calcific mice at 4 and 16 weeks in **a** leaflets, **b** commissures and **c** root. Average A_860/525_ intensities of control and pro-calcific mice at 4 and 16 weeks in **d** leaflets, **e** commissures and **f** root. Average A_810/460_ intensities of control and pro-calcific mice at 4 and 16 weeks in **g** leaflets, **h** commissures and **i** root. Average A_860/525_ intensities of control and pro-calcific mice at 4 and 16 weeks in **j** leaflets, **k** commissures and **l** root. N = 4. *p < 0 .05. TPEF intensity values: Two-way ANOVA with Tukey HSD post hoc test

The A_755/460_ and A_860/525_ autofluorescence intensities did not show significant differences in the leaflets (Fig. 4a, d), commissures (Fig. 4b, e), and roots (Fig. 4c, f) (all p > 0.1) for control and pro-calcific mice at 4 and 16 weeks. The A_755/460_ intensity tended to increase at the commissures of the 16-week pro-calcific mice compared to the 16-week control mice (p = 0.0597) and 4-week pro- calcific mice (p = 0.0806). The A_755/460_ intensity also tended to increase at the roots of the 16-week pro-calcific mice compared to the 16-week control mice (p = 0.0869). The A_810/460_ (p > 0.1) and A_810/525_ (p > 0.1) autofluorescence did not change in the leaflets (Fig. 4g, j). The A_810/460_ intensity increased significantly at the commissures of the 16-week pro-calcific mice compared to the 16-week control mice (p = 0.0115) and 4-week pro—calcific mice (p = 0.0304) and also increased with respect to 4-week control mice (p = 0.0514) (Fig. 4h). The A_810/525_ intensity increased significantly at the commissures of the 16-week pro-calcific mice compared to the 16-week control mice (p = 0.0174) (Fig. 4k). The A_810/460_ intensity increased significantly at the roots of the 16-week pro-calcific mice compared to the 16-week control mice (p = 0.0045) and 4-week pro- calcific mice (p = 0.0154) and also increased with respect to 4-week control mice (p = 0.0775) (Fig. 4i). The A_810/525_ intensity increased significantly at the roots of the 16-week pro-calcific mice compared to the 16-week control mice (p = 0.0059) and 4-week pro-calcific mice (p = 0.0463) (Fig. 4l). The A_810/460_ intensity correlated significantly with the A_755/460_ intensity for commissures (R = 0.9482, p < 0.0001) and roots (R = 0.6938, p = 0.0029) but not for leaflets (R = 0.2028, p = 0.4512). The A_810/525_ intensity correlated significantly with the A_860/525_ intensity for commissures (R = 0.8644, p < 0.0001) and roots (R = 0.6501, p = 0.0064) but not for leaflets (R = 0.0302, p = 0.9117).

### TPEF autofluorescence ratios altered with calcific disease progression in murine aortic valves

**Fig. 5:**
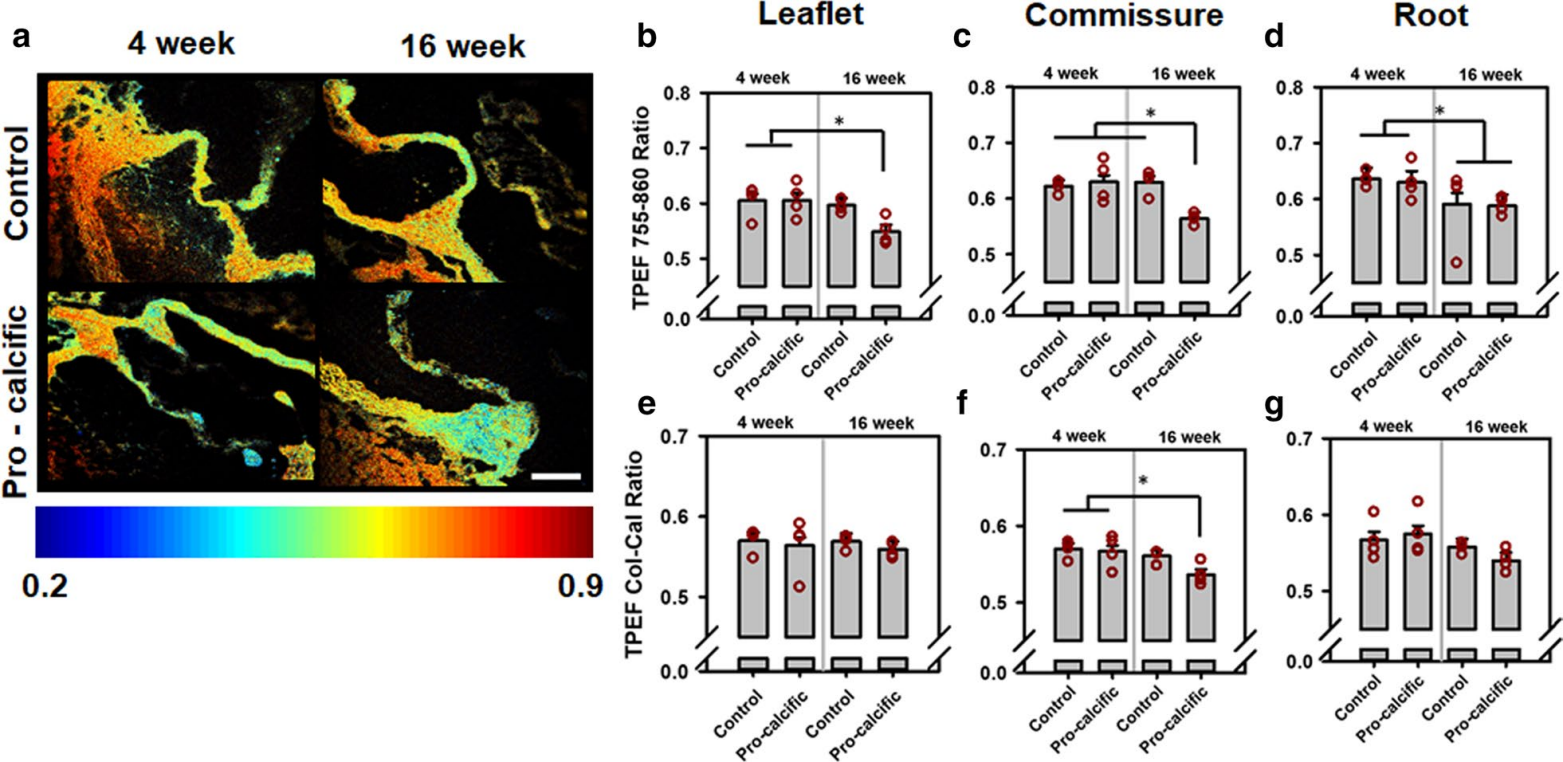
Assessment of TPEF Autofluorescence Ratios via TPEF imaging. **a** Representative TPEF autofluorescence ratio maps for aortic valves of control and pro-calcific mice at 4 and 16 weeks. TPEF 755–860 ratio for **b** leaflets, **c** commissures and the **d** root of control and pro-calcific mice at 4 and 16 weeks. TPEF Col-Cal ratio for **e** leaflets, **f** commissures and the **g** root of control and pro-calcific mice at 4 and 16 weeks. Scale bars—100 µm. N = 3–4. *p < 0.05. Two-way ANOVA with Tukey HSD post hoc test

Using the above autofluorescence intensities, we first obtained TPEF 755–860 ratio maps (Fig. 5a). Mouse valve sections from the control treatment group, without positive ARS staining and without positive expression for the osteogenic markers RUNX2 and osteopontin, and pro-calcific group mouse valves with positive ARS staining and positive expression for these same osteogenic markers, were considered for analysis. The TPEF 755–860 ratio at the leaflets of 16-week pro-calcific mice was significantly lower than 4-week control (p = 0.035) and pro-calcific mice (p = 0.033) and also lower than 16-week control mice (p = 0.0768) (Fig. 5b). The TPEF 755–860 ratio at the commissures (Fig. 5c) in the pro-calcific mice at 16 weeks was significantly lower, when compared to the 16-week control (p = 0.0075), 4-week control (p = 0.0175) and pro-calcific mice (p = 0.0069), also coinciding with positive ARS and RUNX expression. The TPEF 755–860 ratio decreased significantly at the roots (Fig. 5d) (p = 0.0492) at 16 weeks compared to the 4-week mice.

The TPEF Col-Cal ratio was assessed to evaluate differences in the relative contribution of collagen and calcium autofluorescence [29]. The TPEF Col-Cal ratio at the commissures decreased significantly in the 16-week pro-calcific mice when compared to 4-week control mice (p = 0.0303) and pro-calcific mice (p = 0.0498), but did not change with respect to 16-week control mice (p = 0.1375) (Fig. 5f). The TPEF Col-Cal ratio did not show any differences between treatment groups in the leaflets (p > 0.1) (Fig. 5e) and roots (p > 0.1) (Fig. 5g). Given both TPEF autofluorescence ratios may be influenced by collagen, lipids, mineralized calcific deposits, NADH, and FAD [29, 30], we sought to assess the if TPEF Col-Cal and 755–860 ratio were correlated. However, there was no significant correlation observed between the TPEF Col-Cal ratio and the TPEF 755–860 ratio in leaflets (R = 0.1928, p = 0.4743) (Additional file 1: Fig. 4A), commissures (R = 0.3473, p = 0.1876) (Additional file 1: Fig. 4B) and roots (R = 0.4522, p = 0.0786) (Additional file 1: Fig. 4C).

### TPEF autofluorescence ratios negatively correlated with increased calcific disease progression in murine aortic valves

**Fig. 6:**
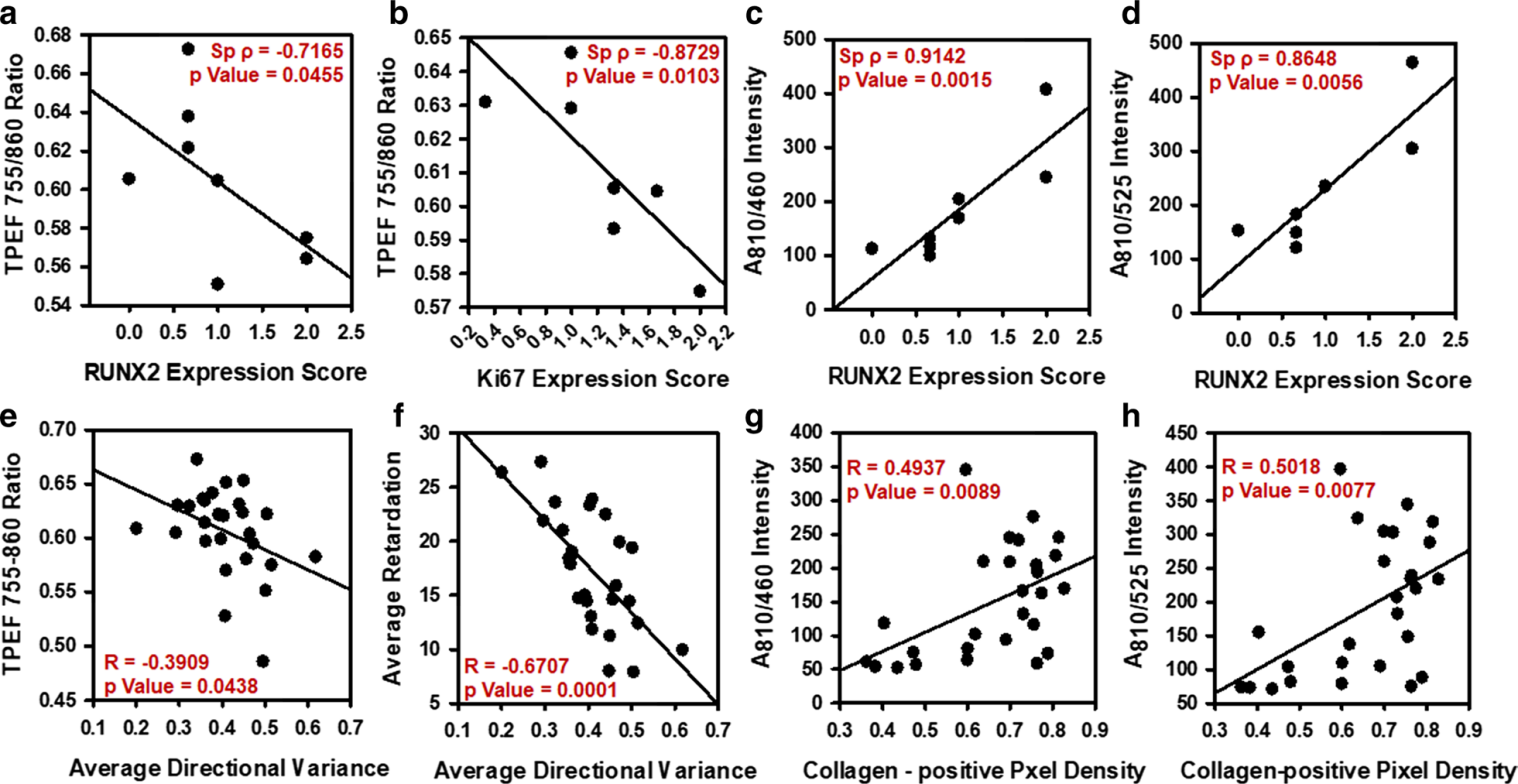
Correlation of TPEF Autofluorescence Ratios with Collagen remodeling and Osteogenic Protein Expression. TPEF 755–860 Ratio for commissures correlated with **a** RUNX2 expression and **b** Ki67 Expression score in control and pro-calcific mice for 4 and 16 weeks. **c** Average A_810/460_ intensity and **d** Average A_860/525_ intensity correlated with RUNX2 expression score in control and pro calcific mice for 4 and 16 weeks. **e** TPEF 755–860 Ratio correlated with average directional variance of collagen from leaflets, commissures and roots of control and pro calcific mice for 4 and 16 weeks. **f** Average retardation correlated with average directional variance of collagen from leaflets, commissures and roots of control and pro calcific mice for 4 and 16 weeks. **g** Average A_810/460_ intensity and **h** Average A_860/525_ intensity correlated with Collagen-positive pixel density from leaflets, commissures and roots of control and pro calcific mice for 4 and 16 weeks. N = 1–3. *p < 0.05. Correlations between TPEF Autofluorescence Ratios and Protein expression score: Spearman’s Rank Correlation; Correlations between TPEF Autofluorescence Ratios and Collagen remodeling metrics: Pearson’s correlation

To quantify the relationship between TPEF autofluorescence ratio alterations and increased disease progression, TPEF autofluorescence ratios were correlated with immunohistochemistry scores and collagen-remodeling metrics obtained via QPLI. The TPEF 755–860 ratio correlated negatively with RUNX2 expression (Spearman’s ρ = −0.7165, p = 0.0455) (Fig. 6a) and Ki67 expression (Spearman’s ρ = −0.8729, p = 0.0103) (Fig. 6b), suggesting pro-calcific mice had increased RUNX2 and Ki67 expression with decreased TPEF 755–860 ratio at the commissures. The A_810/460_ (Fig. 6c) and A_810/525_ (Fig. 6d) intensities also positively correlated with RUNX2 expression (Spearman’s ρ = 0.9142, p = 0.0015 and Spearman’s ρ = 0.8648, p = 0.0056, respectively) at the commissures, suggesting RUNX2-expressing diseased areas may exhibit higher collagen and calcium deposition.

The TPEF 755–860 ratio also correlated positively with average retardation (R = 0.5272, p = 0.0434) for pro-calcific mice and negatively with average directional variance (R = −0.3909, p = 0.0438) (Fig. 6e), while average retardation correlated negatively with average directional variance (R = −0.6707, p = 0.0001) (Fig. 6f). At 4 weeks, TPEF Col-Cal ratio also correlated negatively with retardation (R = −0.6139, p = 0.0067) suggesting decrease in Col–Cal ratio coincided with increased retardation and thicker collagen fibers. At 4 weeks, slightly thicker fibers were found in pro-calcific mice. We additionally found that at 16 weeks, A_810/460_ and A_810/525_ intensities also positively correlated with average local variance (R = 0.7458, p = 0.021 and R = 0.728, p = 0.0262, respectively). Further, A_810/460_ (Fig. 6g) and A_810/525_ (Fig. 6h) intensities positively correlated with collagen positive pixel density (R = 0.4937, p = 0.0089 and R = 0.5018, p = 0.0077, respectively).

### Lower 755–860 Ratio may be predictive of increased calcification

**Fig. 7:**
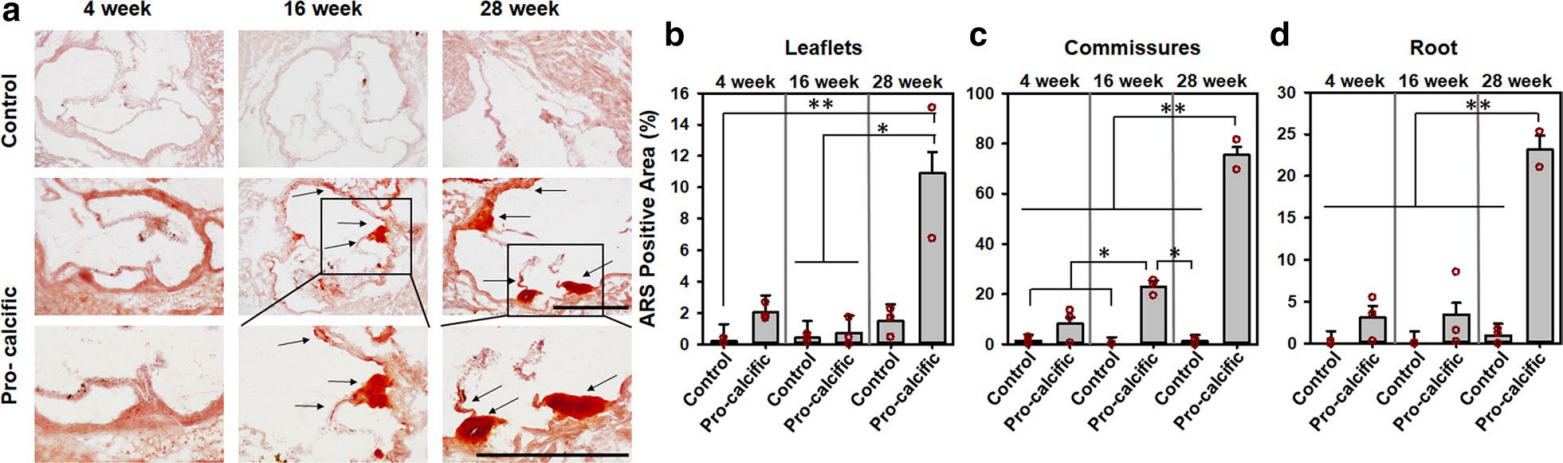
Assessment of Alizarin Red S Positive Area. **a** Alizarin Red S (ARS) stained transverse sections of aortic valves of control and pro calcific mice from 4, 16 and 28 weeks. Mean percentage area covered by ARS positive region of the **b** leaflets, **c** commissures and **d** root of aortic valves of control and pro calcific mice from 4, 16 and 28 weeks. Scale bars = 500 µm. N = 2–3. **p < 0.0001, *p < 0.05. Two-way ANOVA with Tukey’s HSD post-hoc multiple comparisons

To assess if presentation of lower 755–860 ratio preceded increased calcification, the custom diet was continued to be fed to mice until week 28, in a separate trial. Control and pro-calcific mice at 28 weeks were assessed for calcification via ARS staining (Fig. 7a) and ARS positive percentage area was quantified for leaflets, commissures, and root. It was observed that the leaflets of pro-calcific mice at 28 weeks showed significantly increased ARS positive calcification as compared to 16-week control (p = 0.0393), pro-calcific (p = 0.0305) mice and 4-week control mice (p = 0.0113) (Fig. 7b). It was also observed that the commissures (Fig. 7c) and roots (Fig. 7d) of pro-calcific mice at 28 weeks showed significantly increased ARS positive calcification as compared to 4- and 16-week mice and 28-week control mice (p < 0.0001). Additionally, commissures of pro-calcific mice at 16 weeks showed significantly increased ARS positive calcification as compared to 4-week mice (p < 0.01) and 16 (p = 0.0004) and 28 (p = 0.0006) week control mice (Fig. 7c). Indeed, the ARS positive percentage area correlated negatively with TPEF 755–860 ratio for commissures (Spearman’s ρ = -0.8857, p = 0.0188) and roots Spearman’s ρ = -0.9429, p = 0.0048) suggesting a decreasing TPEF 755–860 ratio could indicate increasing calcification.

## Discussion

The most common small animal model for CAVD is the hypercholesterolemic mouse with apoE and/or low density lipoprotein receptor knockouts [14]. Other approaches involve genetically-modified mouse models with NOTCH1 ^+/−^ (NOTCH1 heterozygous), Postn^−/−^ (periostin deletion), or NOS3^−/−^ (endothelial nitric oxide synthase deletion) phenotypes [14, 46]. These genetic mouse models are typically combined with a high fat and high cholesterol western diet [14, 46]. Most of these models have been shown to develop valve calcification in aged mice, or require twenty weeks or more of treatment [41, 47]. Another prior CAVD mouse model was created using a guidewire inflicted aortic valve tissue injury and was shown to develop calcification by twelve weeks following injury [41]. In the current study, we developed and validated a diet-based wild-type small animal model for early progression of valve calcification, without genetic manipulation which showed early hallmarks of CAVD by 4 and 16 weeks. Male mice were chosen due to the higher prevalence of CAVD in males [48]. We observed positive ARS staining of calcification at the commissural walls, similar to what Assmann et al. observed in a rat model [18]. However, unlike the rat model in Assmann et al*.* we did not observe evidence of calcification in the aortic roots.

The mice in the pro-calcific diet groups also had evidence of lipid deposition near the commissural walls at 4 weeks as has been previously reported in mice on a high-fat diet with added cholesterol [14]. Two previous studies reported valve mineralization in wild-type diet-based mouse models without genetic manipulation approximately 16 weeks after initiation of diet [16, 17]. However, the diet employed in these studies incorporated 58.7% fat, which is significantly more than the western diet, and also contained additional carbohydrates. In addition, demonstration of CAVD in these earlier studies relied solely on positive von Kossa particulate staining, which can be confounded in a mouse model by melanocytes or lipofuscin-containing granules with appearance similar to that of von Kossa positive calcification [17]. In our model, we have shown positive ARS staining concurrent with the location of increased activation, proliferation and osteogenesis, suggesting improved utility of our model [14]. To our knowledge, this is the only wild-type diet-based mouse model for early CAVD that has shown ARS-positive calcium staining.

Subtle increases in TGFβ1, which have been known to influence VIC proliferation, differentiation and apoptosis, were observed in the pro-calcific mice at 4 and 16 weeks. This observation, together with increased Ki67, osteopontin, and RUNX2 expression suggest increased proliferation and osteogenic differentiation are occurring in the tissues, which are known hallmarks of CAVD [42] and has been previously reported in various other mouse models [5, 11, 14, 17, 18, 41, 46, 47]. Additionally, concurrent expression of TGFβ1, αSMA and Ki67 also suggests that increased TGFβ1 could have led to the increased proliferation of the myofibroblastic phenotype of VICs in our disease model [49].

Via QPLI, we observed that the 4-week pro-calcific mice had more mature collagen fibers and a higher positive pixel density with minimal changes in collagen fiber orientation compared to controls, suggesting increased collagen remodeling [14, 45, 47]. In the 16-week pro-calcific mice, we observed a decrease in collagen thickness and density with an increased variance in collagen orientation in the leaflets. This was corroborated by the negative correlation between average retardation and average local directional variance. This led us to speculate that by 16 weeks, there was either greater collagen degradation or de novo collagen synthesis, leading to thinner fibers and increased variance in fiber orientation. Collagen remodeling is typically regulated by multiple factors including matrix metalloproteinases (MMPs), cathepsins or other pro-fibrotic factors like TGFβ and twist-1 [50]. In fact, increased MMP activity co-localized with calcification at the commissures in rats fed with a similar diet in other studies [18]. ECM changes have been associated with changes in phenotype [51]. Moreover, in our study, the pro-calcific mice demonstrated a more myofibroblastic phenotype, as evidenced by increased expression of αSMA, which could further contribute towards matrix remodeling. Pro-calcific mice also had a higher percentage of matured collagen fibers in diseased regions at 4 weeks, consistent with CAVD progression [14, 45, 47].

Previously, TPEF microscopy has been used to demonstrate collagen remodeling through SHG imaging [45] and calcification through autofluorescence imaging [29] in the valve at the tissue level. TPEF microscopy can also be used to obtain an optical redox ratio ([FAD]/([FAD] + [NADH]) which reflects the metabolic state of the cell [13, 27, 39]. It has been shown in vitro that mesenchymal stem cells undergoing osteogenic differentiation exhibit decreased optical redox ratios [27]. We have also shown previously that VICs under pathological stretch have reduced optical redox ratios compared to unstretched cells [13]. We have also demonstrated that when VICs are subjected to osteogenic conditions in vitro, their optical redox ratio decreased and correlated with gene expression of osteogenic markers like RUNX2, osteopontin and osteocalcin [28]. In the current study, we measured the TPEF 755–860 ratio (A_860/525_/(A_755/460_ + A_860/525_)) from ex vivo tissue sections by obtaining the autofluorescence intensities at 755 nm and 860 nm excitation, which correspond to the NADH and FAD excitation wavelengths. However, we did not term it an optical redox ratio, as tissue autofluorescence measured via the TPEF 755–860 ratio can be attributable to multiple molecules including collagen, lipids, and mineralized calcific deposits [29, 30]. The TPEF 755–860 ratio could thus have been influenced by collagen and calcium in the tissue [29]. We therefore assessed the TPEF Col-Cal ratio, where the individual A_810/460_ and A_810/525_ intensities correspond to collagen and calcium, respectively [29]. The TPEF Col-Cal ratio increased significantly in aortic valve roots and commissures for the 16-week pro-calcific mice suggesting an increase in collagen and calcium autofluorescence.

In our study, we found that the TPEF autofluorescence ratio rather than the TPEF Col-Cal ratio was more sensitive to disease progression. While TPEF Col-Cal ratio did not show statistical significance between control versus pro-calcific mice, TPEF 755–860 ratio was sensitive to both time, presence of disease conditions, and their interaction. TPEF Col-Cal ratio only showed differences in the commissures, while TPEF 755–860 ratio showed significant changes in the leaflets, commissures and roots. We speculate the difference in sensitivity between TPEF 755–860 and TPEF Col-Cal ratios might be because of increased sensitivity of the TPEF 755–860 ratio to NADH. It should be noted, however, that both these TPEF autofluorescence ratios are influenced by collagen, lipids, mineralized calcific deposits, NADH, and FAD [29, 30].

Additionally, we observed that TPEF 755–860 ratio correlated with RUNX2 and Ki67 negatively suggesting increased osteogenesis and proliferation was marked by reduced TPEF 755–860 ratio, which is consistent with previous studies [13, 27, 32, 39, 52]. It is also interesting to note that A_810/460_ and A_810/525_ intensities positively correlated with RUNX2 expression leading us to believe that RUNX2-expressing diseased areas may exhibit higher collagen and calcium deposition. Indeed, A_810/460_ and A_810/525_ intensities also positively correlated with average directional variance and collagen positive pixel density. This suggested that the increase in A_810/460_ and A_810/525_ intensities indeed allude to increased collagen [45] and calcium [29] deposition during disease progression. TPEF 755–860 ratio which showed a decrease during disease progression correlated negatively with average directional variance further evidencing, thinner disorganized fibers during disease progression. This may suggest increased de novo collagen and calcium deposition with increased CAVD progression. Similar correlation between cell redox state and collagen synthesis is consistent with other studies [27, 53, 54]. The ratio of blue:green (i.e. 460 nm:525 nm emission) TPEF autofluorescence at 800 nm excitation has been shown to be sensitive to mineralization and altered CAVD progression [29]. Our current study is the first instance when a TPEF 755–860 ratio has been shown to demonstrate tissue level changes occurring during early CAVD progression in a mouse model that was more sensitive than the TPEF Col-Cal ratio. Like we had previously demonstrated in our in vitro studies [13, 28, 52], in this study also we observed TPEF autofluorescence ratios decreased at 16 weeks in the pro-calcific mice commissures and correlated with increased calcification, osteogenic differentiation and proliferation. This is also similar to the findings of Quinn et al*.* where the osteogenic differentiation of the mesenchymal stem cells correlated with the reduction in an optical redox ratio [27] and Jones et al. where increased proliferation correlated with a reduction in redox ratio in skin wound healing [39]. TPEF 755–860 ratio not only correlated with osteogenic progression and increased proliferation but also seemed to have a predictive quality for calcification. This further provides an incentive to explore TPEF 755–860 as an early biomarker for CAVD progression.

## Limitations

One limitation of this study was that the autofluorescence signal from various endogenous fluorophores like NADH, FAD, lipids, collagen, elastin, and mineralization that are likely present in valves [29, 32] was not decoupled. Additionally, this study was conducted ex vivo, considering the limitations of current TPEF imaging probes to assess cardiac valves. Although intravital pericardial imaging has been a possibility in recent past [55–57], clinical translation of TPEF imaging in valves is yet to be realized. Enhanced probe flexibility and miniaturization might open avenues for pre-clinical studies on larger animal models in the future [58, 59]. Finally, we acknowledge that the sample size for our quantitative analyses was low. However, it should be noted that our effect sizes were sufficient to reach significant conclusions.

## Future directions

Decoupling the signals from various endogenous fluorophores (e.g. NADH, FAD, lipids, collagen, elastin, and mineralization) that are likely present in valves could provide a detailed quantitative assessment of different aspects of disease progression [29, 32]. More robust studies incorporating correlation between the TPEF metrics and established metrics of disease progression both in vivo and in vitro, would further demonstrate the utility of TPEF metric to track disease progression. However, our study provides sufficient evidence to suggest that the TPEF 755–860 autofluorescence ratio can be further explored to serve as a label-free metric for assessing CAVD progression.

## Conclusions

In conclusion, we created a wild-type mouse model for early CAVD where the mice showed lipid deposition and collagen remodeling at 4 weeks and early evidence for calcification at 16 weeks. The valvular apparatus showed signs of proliferation, osteogenic differentiation, and increased inflammatory cytokine infiltration. The diseased valves also showed altered TPEF autofluorescence signatures, which were consistent with the findings in previous studies [27–29] and correlated with osteogenic progression, ECM remodeling and calcification Additionally, 28-week ARS stains revealed presence of calcification in leaflets that was previously absent until 16 weeks. Enhanced calcification in the commissures and roots and negative correlation of TPEF 755–860 ratio with ARS positive calcification suggests lower autofluorescence ratios may be potentially explored as a predictive biomarker for calcification. This study suggests that with further validation, and imaging probe miniaturization, quantitative TPEF metrics hold promise to serve as a label-free tool to assess the progression of CAVD.

## Supplementary information

**Additional file 1**. Supplemental methods and results.

## Data Availability

The data generated and/or analyzed during the current study are included in this article and supplementary information and also available from the corresponding author on reasonable request.

## Abbreviations

CAVD: Calcific aortic valve disease
VEC: Valve endothelial cell
VIC: Valve interstitial cell
ECM: Extracellular Matrix
TPEF: Two-photon excited fluorescence
SHG: Second harmonic generation
FAD: Flavin adenine dinucleotide
NADH: Nicotinamide adenine dinucleotide reduced
QPLI: Quantitative polarized light imaging
PSR: Picrosirius red
ARS: Alizaring Red S staining
EF: Ejection fraction
CO: Cardiac output
EDV: End-diastolic volume
ESV: End-systolic volume
LVIDs: Left ventricle internal diameter systolic
LVIDd: Left ventricle internal diameter diastolic
H & E: Hematoxylin and eosin
α-SMA: Alpha smooth muscle actin
BMP4: Bone morphogenic protein 4
TGFβ1: Transforming growth factor beta 1
MMP: Matrix metalloproteinase
ANOVA: Analysis of variance
SEM: Standard error of the mean
