# Supplementary material for: Label-free optical biomarkers detect early calcific aortic valve disease in a wild-type mouse model
Source: BMC Cardiovasc Disord. 2020 Dec 11;20:521. doi: 10.1186/s12872-020-01776-8 (PMC7731510; doi:10.1186/s12872-020-01776-8)
Supplement: Supplementary file 1 — Additional file 1. Supplemental methods and results. [file 12872_2020_1776_MOESM1_ESM.docx]

**SUPPLEMENTAL MATERIAL**

**SUPPLEMENTAL RESULTS**

**Mice weight in response to the diet**

The mice (Supplemental Fig. 5A) were allowed to feed *ad libidum* and their food intake and weight gain were monitored every three to four days. The amount of food consumed was statistically similar between the control and pro-calcific group (mice on pro-calcific diet) for 16 weeks (Supplemental Fig. 5B). and for 28 weeks (Supplemental Fig. 6A). While the mice weight range was within acceptable levels for all treatment groups within this study, control mice had significantly higher weights, compared to pro-calcific mice for a given time bin up to 16 weeks (Supplemental Fig. 5C) and 28 weeks (Supplemental Fig. 6B).

**Mice on pro-calcific diet did not show altered left ventricular function**

To assess left ventricular function in terms of ejection fraction and cardiac output, echocardiography was performed every 4 weeks. The average ejection fraction (Supplemental Fig. 5D) averaged between 60 to 80% for the control and pro-calcific mice throughout the study. The average ejection fraction was 74.39 ± 2.89% for the control mice and 69.46 ± 2.70% for the pro-calcific mice at the end of the 16-week study duration. There were no significant differences observed in the ejection fraction (p > 0.05) (Supplemental Fig. 5D), cardiac output (p > 0.05) fraction (Supplemental Fig. 5E), end-diastolic volume (p > 0.1) fraction (Supplemental Fig. 5F), and end-systolic volume (p > 0.1) fraction (Supplemental Fig. 5G) between treatment groups at all time points. Even at 28 weeks, there were no significant differences found in ejection fraction (p > 0.05) (Supplemental Fig. 6C), cardiac output (p > 0.05) fraction (Supplemental Fig. 6D), end-diastolic volume (p > 0.1) fraction (Supplemental Fig. 6E), and end-systolic volume (p > 0.1) fraction (Supplemental Fig. 6F) between control and pro-calcific mice.

**Mice plasma levels of calcium and cholesterol in response to diet**

Plasma collected after euthanizing the mice was collected and stored at -80˚C freezer. Calcium assay kit (BioAssay systems, Hayword, CA) was used to quantify plasma calcium levels. Calcium levels in 16-week mice plasma were significantly higher than 4-week mice plasma samples (p < 0.0001) (Supplemental Fig. 7A). Interestingly, plasma calcium levels showed a significant increase in pro-calcific mice as compared to control mice at 4 weeks (p = 0.0115) but not at 16 weeks. High-density lipoprotein (HDL) fraction, low and very low-density lipoprotein (LDL/VLDL) fraction, total and free cholesterol were quantified using cholesterol assay kit (Abcam, Cambridge, MA). While LDL fraction (Supplemental Fig. 7B) did not show any significant differences and HDL fraction (Supplemental Fig. 7C) was higher in 16-week pro-calcific mice as compared to 4 week control mice (p = 0.0451), total (p = 0.0001) (Supplemental Fig. 7D) and free (p < 0.0001) (Supplemental Fig. 7E) cholesterol were found to be significantly higher in 16-week mice plasma. Cholesterol esters (Supplemental Fig. 7F) quantified as the difference between total and free cholesterol also did not show significant difference.

**Variability within TPEF Autofluorescence Ratios**

The variability between the different fields-of-view (FOV) was less than 10% of the TPEF 755-860 ratios (Supplemental Fig. 4E) and TPEF Col-Cal ratios (Supplemental Fig. 4F). The FOV-FOV variability in TPEF 755-860 ratio was found to be 0.045 for the non-calcified region and 0.043 for the calcified region, which is less than 10% of the obtained TPEF 755-860 ratio values. The FOV-FOV variability in TPEF Col-Cal ratio was found to be 0.011 for the non-calcified region and 0.012 for the calcified region, which is less than 10% of the obtained TPEF Col-Cal ratio values.

**
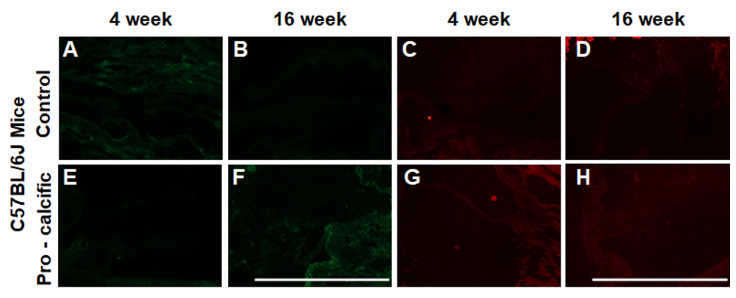
**

**Supplemental Fig 1. No Primary Antibody – Negative Controls Assessed by Immunohistochemistry.** Representative transverse sections of aortic valve from normal and pro-calcific mice at 4 weeks showing no primary negative controls for Alexa fluor-488 (A, B, E, F) and -594 (C, D, G, H) conjugated secondary antibodies in 4 week and 16 week normal and Pro-calcific mice. Scale Bars - 500µm. N = 2-3 (mice).

**
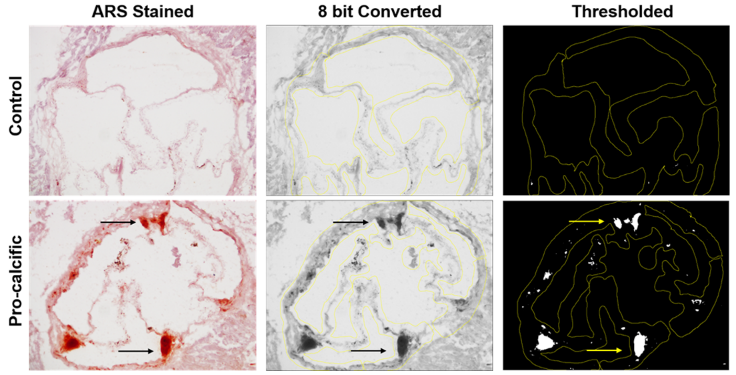
**

**Supplemental Figure 2. Schematic of Assessment of Alizarin red S positive regions.** ARS Stained images were uploaded in image J and converted to 8-bit image. A uniform threshold was used to select the ARS positive regions (marked by arrows). The ImageJ plugin to ‘Analyze Particles’ was utilized to assess the percentage area positive for ARS.

**
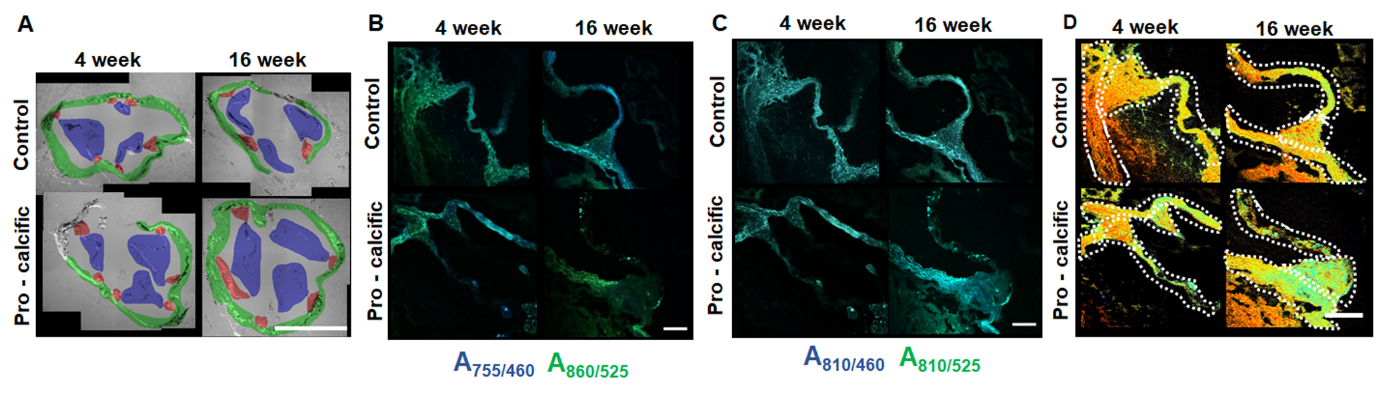
**

**Supplemental Figure 3. Representative Overlay Figures to Supplement Methods.** (A) Maps of the leaflets (blue), commissures (red) and roots (green) used for analyzing the quantitative polarized light imaging metrics. Scale bar - 500µm. (B) Overlay of A_755/460_ intensity map (Blue) and A_860/525_ intensity map (Green) image for control and pro-calcific mice at 4 and 16 weeks. (C) Overlay of A_810/460_ intensity map (Blue) and A_810/525_ intensity map (Green) image for control and pro-calcific mice at 4 and 16 weeks. (D) Outlines for the leaflet, commissures and roots for analyzing the TPEF metrics. Scale bars - 100µm.

**
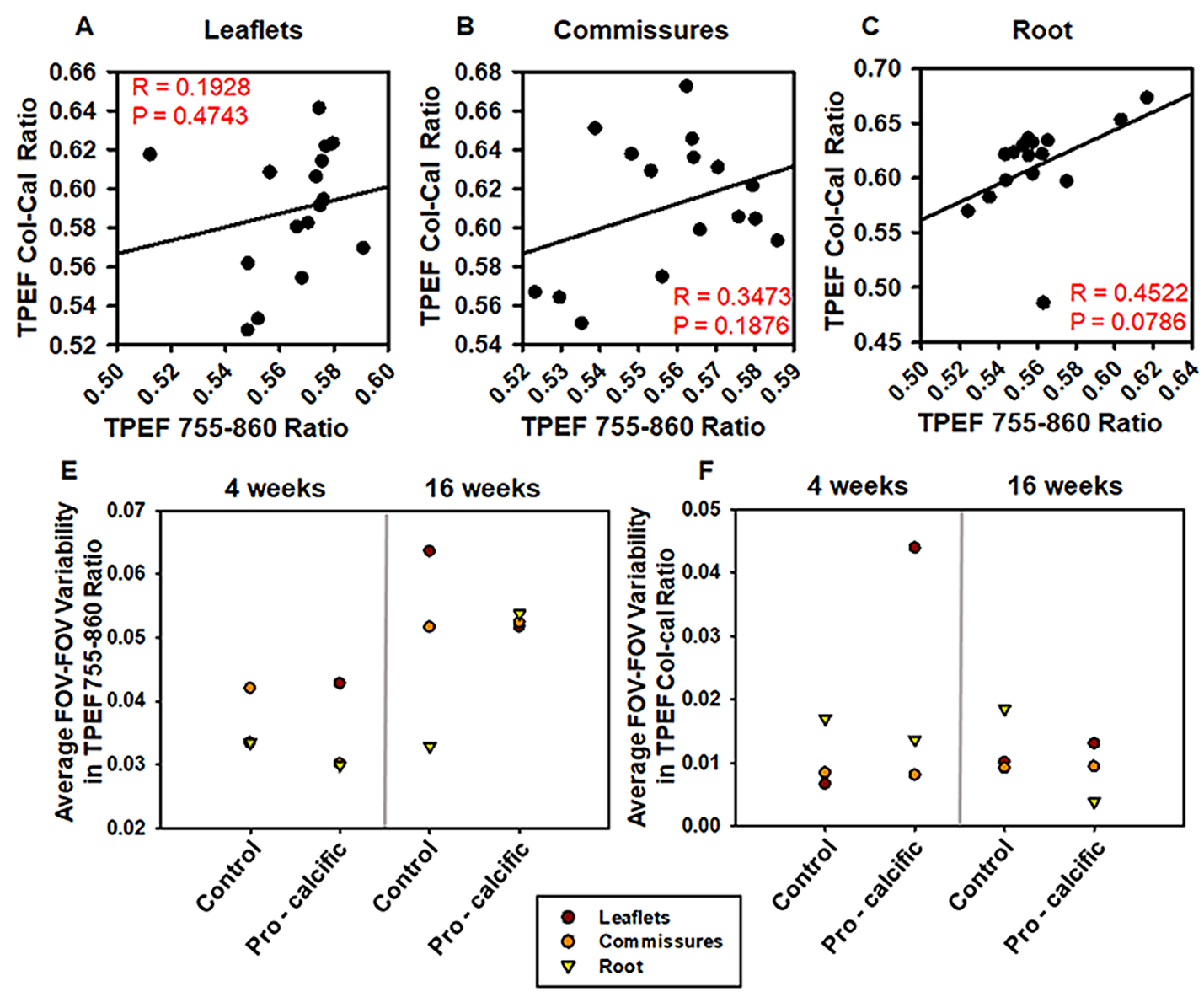
**

**Supplemental Figure 4. Correlation and Variability within TPEF Autofluorescence Ratios.** TPEF Col-Cal ratio did not significantly correlate with TPEF 755-860 ratio for (A) leaflets, (B) commissures and (C) root for the control and pro-calcific mice at 4 and 16 weeks. Average standard deviation within the fields of view (FOV) in (E) TPEF 755-860 ratio and (F) TPEF Col-Cal ratio of leaflets, commissures and roots of control and pro-calcific mice at 4 and 16 weeks. N = 4. Correlation between TPEF Col-Cal Ratio and TPEF 755-860 Ratio: Pearson’s correlation.


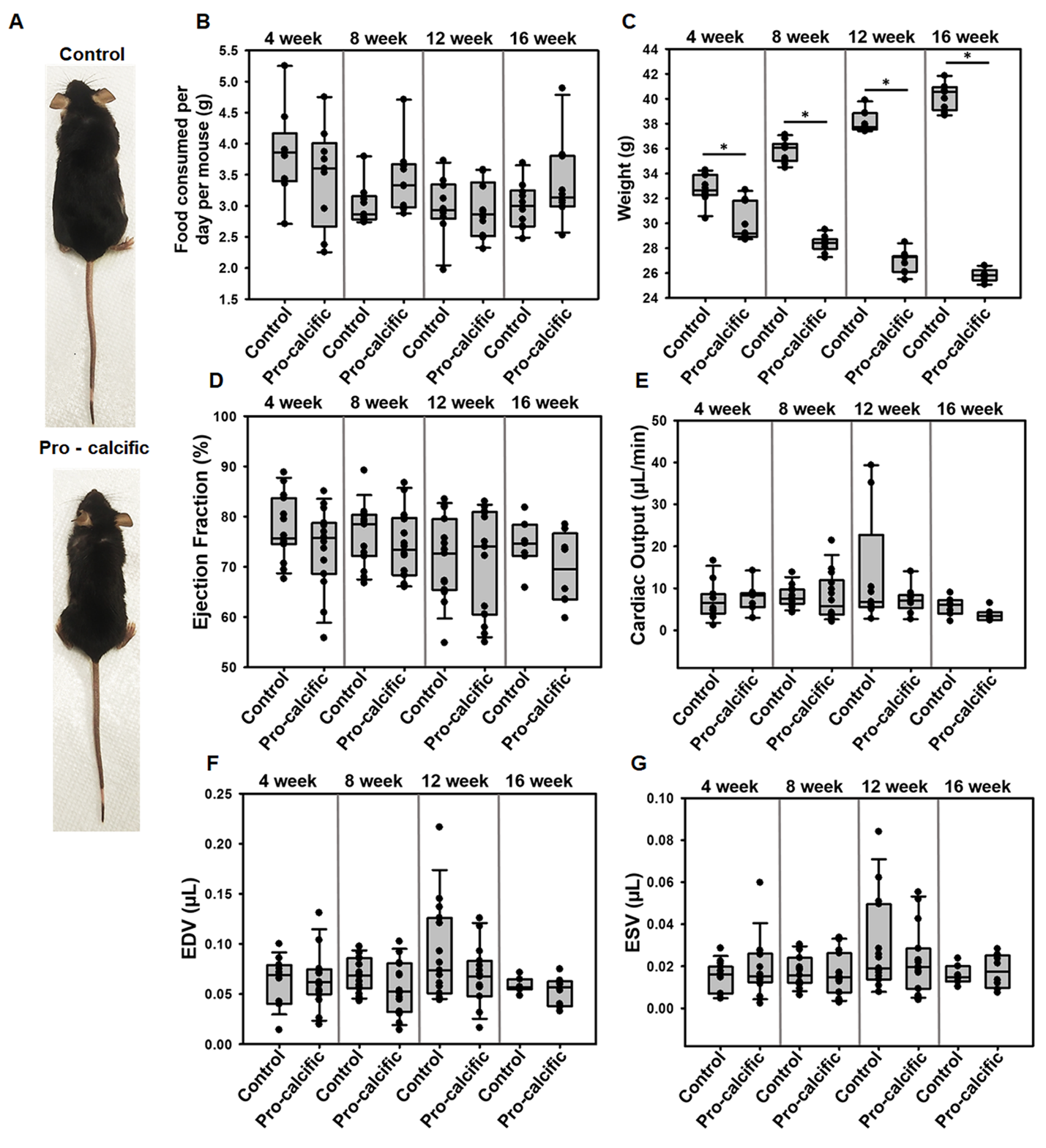


**Supplemental Figure 5. Food Consumption, Weight Gain and Left Ventricular Function for Control and Pro-Calcific Mice up to 16 weeks.** (A) Representative images of control and pro-calcific mice at 16 weeks. (B) Amount of food in grams consumed by the mice per day. ANOVA on Ranks (non-normal data distribution); (C) Weight of mice in grams in response to the control and pro-calcific diet. * p < 0.001. Two-way ANOVA with Holm-Sidak post-hoc multiple comparisons. Control: N = 15-24; Pro-calcific: N=9-10. (D) Mean ejection fraction, (E) cardiac output, (F) end diastolic volume (EDV), and (G) end systolic volume (ESV) at 4, 8, 12 and 16 weeks for control and pro-calcific mice. N > 7, p > 0.05. EF: Two-way ANOVA; EDV, ESV and CO: ANOVA on Ranks (non-normal data distribution).

**
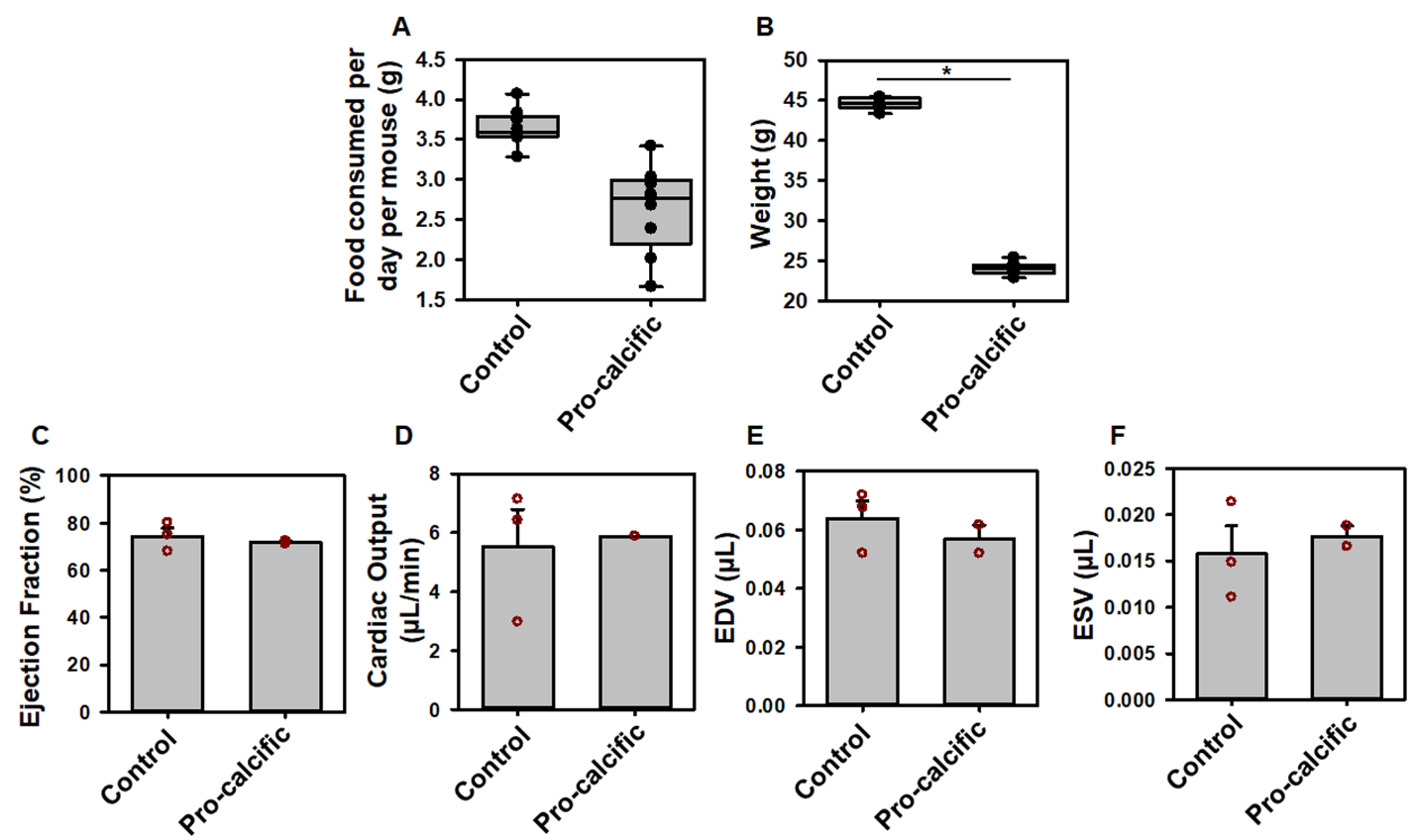
Supplemental Figure 6. Food Consumption, Weight Gain and Left Ventricular Function for Control and Pro-Calcific Mice at 28 Weeks.** (A) Amount of food in grams consumed by the mice per day. ANOVA on Ranks (non-normal data distribution) (B) Weight of mice in grams in response to the control and pro-calcific diet. * p < 0.001. Two-way ANOVA with Holm-Sidak post-hoc multiple comparisons. (C) Mean ejection fraction, (D) cardiac output, (E) end diastolic volume (EDV), and (F) end systolic volume (ESV) at 28 weeks for control (N = 3) and pro-calcific mice (N =2), p > 0.05. ANOVA on ranks (non-normal data distribution).

**
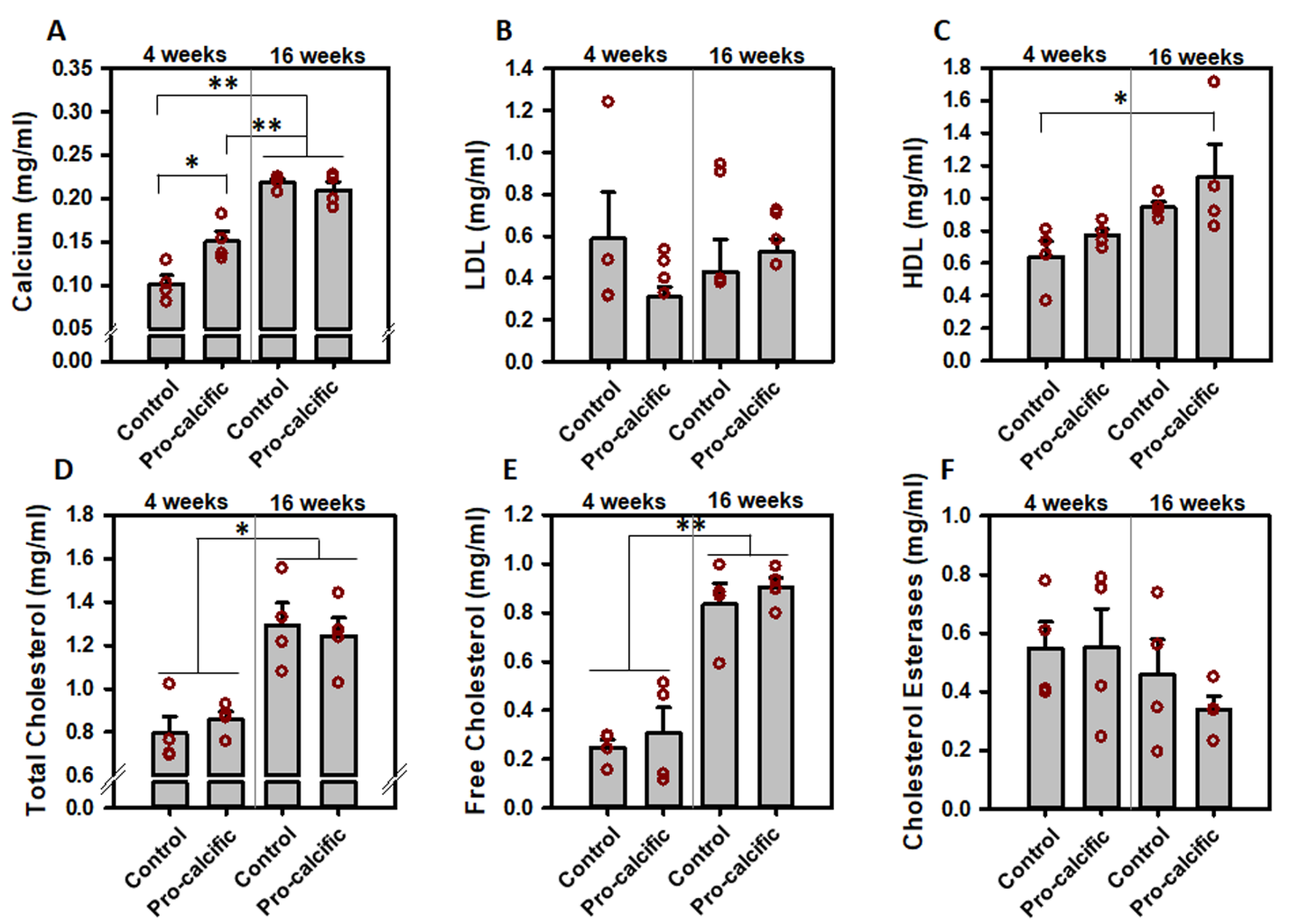
Supplemental Figure 7.** **Mice Plasma levels of Cholesterol and Calcium** (A) Mean calcium level in plasma of control and pro calcific mice at 4 and 16 weeks, Mean (B) low and very low-density lipoprotein (LDL/VLDL) fraction, (C) high-density lipoprotein (HDL) fraction, (D) total cholesterol (E) free cholesterol and (F) cholesterol esterase for control and pro-calcific mice at 4 and 16 weeks. (N = 4), p < 0.05. Two-way ANOVA with Tukey’s HSD post-hoc multiple comparisons.
